# Targeting Pin1 Protects Mouse Cardiomyocytes from High-Dose Alcohol-Induced Apoptosis

**DOI:** 10.1155/2016/4528906

**Published:** 2015-12-01

**Authors:** Yuehong Wang, Zizhuo Li, Yu Zhang, Wei Yang, Jiantao Sun, Lina Shan, Weimin Li

**Affiliations:** ^1^Department of Cardiology, The First Affiliated Hospital of Harbin Medical University, Harbin 150001, China; ^2^Department of Inpatient Abdominal Ultrasonography, The First Affiliated Hospital of Harbin Medical University, Harbin 150001, China; ^3^Department of Neurology, The Fourth Affiliated Hospital of Harbin Medical University, Harbin 150001, China

## Abstract

Long-term heavy alcohol consumption is considered to be one of the main causes of left ventricular dysfunction in alcoholic cardiomyopathy (ACM). As previously suggested, high-dose alcohol induces oxidation stress and apoptosis of cardiomyocytes. However, the underlying mechanisms are yet to be elucidated. In this study, we found that high-dose alcohol treatment stimulated expression and activity of Pin1 in mouse primary cardiomyocytes. While siRNA-mediated knockdown of Pin1 suppressed alcohol-induced mouse cardiomyocyte apoptosis, overexpression of Pin1 further upregulated the numbers of apoptotic mouse cardiomyocytes. We further demonstrated that Pin1 promotes mitochondria oxidative stress and loss of mitochondrial membrane potential but suppresses endothelial nitric oxide synthase (eNOS) expression in the presence of alcohol. Taken together, our results revealed a pivotal role of Pin1 in regulation of alcohol-induced mouse cardiomyocytes apoptosis by promoting reactive oxygen species (ROS) accumulation and repressing eNOS expression, which could be potential therapeutic targets for ACM.

## 1. Introduction

Heart failure continues to be a major public health issue [1]. In the United States, long-term heavy alcohol consumption is the leading cause of nonischemic dilated cardiomyopathy in both genders, known as “alcoholic cardiomyopathy” (ACM) [2, 3]. Generally, patients consuming more than 90 g of alcohol per day for more than 5 years are likely to have asymptomatic ACM, which may develop into symptomatic ACM and signs of heart failure [2, 4].

In the asymptomatic stage, ACM is usually characterized by left ventricular dilation, increased left ventricular mass, and reduced or normal left ventricular wall thickness [3, 5]. Pathologically, previous studies have shown a strong correlation between ACM and cardiomyocyte apoptosis [6]. Apoptotic cardiomyocytes were detected in the heart muscles of individuals with long-term alcoholism, and expression of BAX and BCL-2 was also observed [7, 8]. Studies in animal models also demonstrated that chronic alcohol intake could induce oxidative stress and cellular apoptosis in cardiomyocytes [9, 10]. In a primary cell culture model, alcohol was found to induce reactive oxygen species-mediated apoptosis in a dose-dependent manner in the range of 0–100 mM [8, 11]. However, the molecular mechanism by which alcohol induces apoptosis of cardiomyocytes remains to be investigated.

Peptidyl-prolyl cis-trans isomerase Pin1, a member of the parvulin family of PPIase enzymes, is capable of isomerizing the peptidyl-prolyl bond in specific phosphorylated Ser/Thr-Pro motifs of the substrates, which may lead to profound changes in their activity, stability, phosphorylation status, and protein-protein interactions [12, 13]. Pin1 was originally found to be required for cell division in yeast and human cells. Later studies demonstrated that Pin1 is important for regulation of many other cellular processes, such as gene transcription, cell proliferation, differentiation, and apoptosis [14]. In addition, since phosphorylation of proteins is an essential signaling mechanism, Pin1 is involved in the Ras signaling pathway and activation of Wnt signaling [15, 16].

With regard to regulation of apoptosis, Pin1 was found to inhibit apoptosis in hepatocellular carcinoma cells and SW620 cells in colorectal carcinoma [17, 18]. In this study, we further investigated the role of Pin1 in regulation of high-dose alcohol-induced cardiomyocyte apoptosis and found that alcohol induced Pin1 expression and activation in a dose-dependent manner in primary mouse cardiomyocytes. We further demonstrated that targeting of Pin1 protects cardiomyocytes from high-dose alcohol-induced apoptosis by regulating mitochondria oxidative stress and endothelial nitric oxide synthase (NOS) expression.

## 2. Materials and Methods

### 2.1. Cell Culture, Cell Transfection, and Reagents

Primary cardiomyocytes were isolated from neonatal mouse hearts, as described previously [19]. Briefly, heart tissue was minced and digested, using a collagenase/dispase mixture (Roche, Indianapolis, IN). Tissue fragments were allowed to sediment, and the supernatant-containing suspended cells were preplated for 2 h to remove fibroblasts and endothelial cells. Enriched cardiomyocytes were then cultured in collagen-coated dishes at approximately 1.5 × 10^5^ cells per cm^2^. All animal procedures were conducted in accordance with the Guidelines for the Care and Use of Laboratory Animals at Harbin Medical University and approved by the Chancellor's Animal Research Committee.

Scrambled and Pin1 siRNAs were purchased from Invitrogen (Carlsbad, CA) and transfected with Lipofectamine RNAiMAX (Invitrogen). Pin1 plasmids were obtained from Addgene (Cambridge, MA). Lipofectamine LTX (Invitrogen) was used for plasmid transfection according to the manufacturer's instructions. Cardiomyocytes (5 × 10^4^ cells/well) were seeded onto 24-well plates and grown overnight to approximately 80% confluence. The cells were transfected with 30 pmol siRNA or 500 ng plasmid and incubated for 48 h, and subsequent experiments were performed after transfection to analyze efficiency, using western blotting.

N-acetylcysteine (NAC) and Mito-TEMPO were purchased from Sigma-Aldrich (St. Louis, MO, USA).

### 2.2. Quantitative Reverse Transcription Polymerase Chain Reaction (qRT-PCR)

Total RNA was extracted from cardiomyocytes, using the TRIzol reagent (Life Technologies, Gaithersburg, MD) according to the manufacturer's instructions, and complementary DNA was then synthesized from 1 *μ*g of total RNA from each sample, using the SuperScript III First-Strand Synthesis System (Life Technologies). mRNA expression levels of Pin1 were measured by qRT-PCR and calculated using the 2^−ΔΔCt^ method and normalized to the expression of glyceraldehyde 3-phosphate dehydrogenase (GAPDH). The primers of oligonucleotides were as follows:

 5′-CCGGAATTCATGGCGGACGAGGAGAAG-3′ (forward) and
 5′-TGCTCTAGATCATTCTGTGCGCAGGAT-3′ (reverse) for Pin1;
 5′-TGGACTCCACGACGTACTCAG-3′ (forward) and 5′-GGGAAGCTTGTCATCAATGGAA-3′ (reverse) for GAPDH.

### 2.3. Western Blotting

Cardiomyocytes were harvested and lysed on ice for 30 min in RIPA buffer (120 mM NaCl, 40 mM Tris [pH 8.0], and 0.1% NP 40) with proteinase/phosphatase inhibitor (Pierce, Rockford, IL). The lysates were centrifuged at 18,000 g for 15 min at 4°C. The supernatants were collected, and protein concentrations were determined by the BCA method (Pierce). Aliquots of the lysates were electrophoresed on a 10% sodium dodecyl sulfate-polyacrylamide gel (SDS-PAGE). The resolved protein was then transferred onto nitrocellulose membranes (Bio-Rad, Hercules, CA), which were subsequently incubated with primary antibodies, followed by a horseradish peroxidase-conjugated secondary antibody (Boster, Wuhan, China). Protein bands were detected using an enhanced chemiluminescence western blotting detection kit (Pierce), followed by exposure of the membranes to X-ray film. Primary antibodies for Pin1, cytochrome c (Cyt.C), endothelial nitric oxide synthase (eNOS), mHsp70, p66^shc^, and *β*-actin were purchased from Santa Cruz (Santa Cruz, CA).

### 2.4. Pin1 Activity Assays

Pin1 activity was analyzed as previously described [20, 21]. Briefly, cardiomyocytes were lysed by sonication in lysis buffer (50 mM N-2-hydroxyethylpiperazine-N′-2-ethanesulfonic acid, 100 mM NaCl, 0.25% 3-[(3-cholamidopropyl) dimethylammonio]-1-propanesulfonate (CHAPS), 5 mM NaF, 1 mM *β*-glycerophosphate, and 1 mM ethylene glycol tetraacetic acid) at 4°C. We then prepared a mixture containing 93 *μ*L of N-2-hydroxyethylpiperazine-N′-2-ethanesulfonic acid buffer (50 mM N-2-hydroxyethylpiperazine-N′-2-ethanesulfonic acid (pH 7.8), 100 mM NaCl, 2 mM DTT, 0.04 mg/mL of bovine serum albumin), 5 *μ*L of cell lysate (10^5^ cells or 0.25 nmol of recombinant Pin1), and 2 *μ*L (20 mg/mL) of trypsin solution. The reaction was started by adding 50 *μ*L (720 *μ*M) of peptide Trp-Phe-Tyr-Ser (PO_3_H_2_)-ProArg-pNA (NeoMPS), followed by* p*-nitroaniline absorbance at 390 nm for 4 min.

### 2.5. Cell Viability Assays

Cardiomyocyte viability was measured using a 3-(4,5-dimethylthiazol-2-yl)-2,5-diphenyltetrazolium bromide (MTT) assay. Cells were plated at 3 × 10^3^ cells per well in 100 *μ*L culture medium in 96-well culture plates. Sterile MTT dye (20 *μ*L) (Sigma) was added to each well. After incubating for 4 h at 37°C, MTT medium mixture was removed, and 200 *μ*L of dimethyl sulfoxide (Sigma) was added to each well. The absorbance representing viable cells was measured by a microplate reader at a wavelength of 490 nm. Cell viability was calculated as a relative ratio of the control.

### 2.6. Caspase Activity Assays

Cells (1 × 10^4^) were incubated in 96-well plates for 24 h, and caspase-3 activity was measured using the caspase-3 and caspase-9 assay kits (Ambion, Austin, TX) according to the manufacturer's protocol. Briefly, the cells were incubated with lysis buffer on ice for 10 min and collected via 10,000 g centrifugation for 10 min. Protein (100 *μ*g) from each sample was incubated with specific colorimetric tetrapeptides Asp-Glu-Val-Asp-p-nitroaniline (pNA; specific substrate of caspase-3) or Leu-Glu-His-Asp-pNA (specific substrate of caspase-9) at 37°C for 60 min. The activity of caspase-3 and caspase-9 was quantified using a spectrophotometer at 405 nm, and the data were normalized to the control group.

### 2.7. TUNEL Staining

TUNEL staining was performed using the DeadEnd Fluorometric TUNEL System (Promega, Madison, WI) according to the manufacturer's protocol. Briefly, cells were grown on chambered culture slides, fixed with 4% PFA for 2 h at room temperature, and permeabilized with 0.2% Triton X-100 in PBS for 5 min. Cells were washed again with PBS and equilibrated with 100 *μ*L equilibration buffer at room temperature for 10 min. Slides were covered with 50 *μ*L of terminal deoxynucleotidyl transferase reaction mixture for 60 min in a humidified chamber. The reaction was stopped with 2x SSC for 15 min. Nuclei were visualized by DAPI staining.

### 2.8. Mitochondrial Membrane Potential Measurements

Mitochondrial membrane potential (Δ*ψ*m) was assessed using a TMRE Mitochondrial Membrane Potential Assay Kit (Abcam, Cambridge, England). Cells were incubated with 100 nM TMRE in the absence or presence of CCCP at 20 *μ*M at 37°C for 15 min and washed with 0.2% BSA in PBS. The cell pellet was collected by centrifugation at 1500 g for 3 min and resuspended in 1 mL of PBS. Fluorescence was measured by a fluorescence plate reader (BioTek, Burlington, VT).

### 2.9. Measurement of Cyt.C Release from Mitochondria to Cytosol

Release of Cyt.C from mitochondria to cytosol was measured by western blotting, as previously described [22]. Cells (5 × 10^6^) were collected by trypsin-EDTA (0.5%), followed by two washes with cold PBS and lysed in ice-cold lysis buffer (250 mM sucrose, 1 mM EDTA, 0.05% digitonin, 25 mM Tris (pH 6.8), 1 mM dithiothreitol, 1 *μ*g/mL leupeptin, 1 *μ*g/mL pepstatin, 1 *μ*g/mL aprotinin, 1 mM benzamidine, and 0.1 mM phenylmethylsulfonyl fluoride). Lysates were centrifuged at 12,000 g at 4°C for 3 min to obtain the supernatants for western blot analysis. Protein concentration in the supernatants was measured using BCA protein assay kit (Thermo Fisher Scientific, Canoga Park, CA).

### 2.10. Measurement of NO Production and Mitochondrial ROS

Nitric oxide production was assessed by measuring the levels of oxidized forms (nitrites and nitrates) in samples, using a nitric oxide assay kit (Abcam, Cambridge, UK). Mitochondrial ROS levels were analyzed using the Elite Mitochondrial ROS Activity Assay Kit (eEnzyme, Gaithersburg, MD). Cells were harvested and incubated with 100 *μ*L Elite ROS Deep Red stain solution on 96-well plates for 60 min at 37°C. Fluorescence intensity was measured at EX/EM = 650/675 nm.

### 2.11. Mitochondrial Protein Isolation

Cells were centrifuged at 370 g for 10 min and washed in 10 packed cell volumes of washing buffer (1 mM Tris HCl, pH 7.4, 0.13 M NaCl, 5 mM KCl, and 7.5 mM MgCl_2_) three times. Cells were then resuspended in 6 packed cell volumes of homogenization buffer (10 mM Tris HCl, pH 7.4, 10 mM KCl, 0.15 mM MgCl_2_, 1 mM PMSF, and 1 mM DTT) and homogenized for 10 min on ice. Homogenate was transferred into a conical centrifuge tube containing 1 packed cell volume of 2 M sucrose solution. Unbroken cells, nuclei, and large debris were removed by centrifuging at 1,200 g for 5 min twice. The mitochondria were collected by centrifuging at 7,000 g for 10 min. The mitochondrial pellet was resuspended in 3 packed cell volumes of mitochondrial suspension buffer (10 mM Tris HCl, pH 6.7, 0.15 mM MgCl_2_, 0.25 mM sucrose, 1 mM PMSF, and 1 mM DTT) for further western blot analysis.

### 2.12. Statistical Analyses

All statistical analyses were performed using SPSS 18.0 software (IBM, Chicago, IL). The significance of differences between groups was estimated by Student's *t*-test, *χ* ^2^ test, or one-way analysis of variance (ANOVA). The data were expressed as the mean ± SEM of three independent experiments. A *p* value of 0.05 or less was considered to be statistically significant.

## 3. Results

### 3.1. Alcohol Induced Pin1 Expression and Activation in Cardiomyocytes

**Figure 1:**
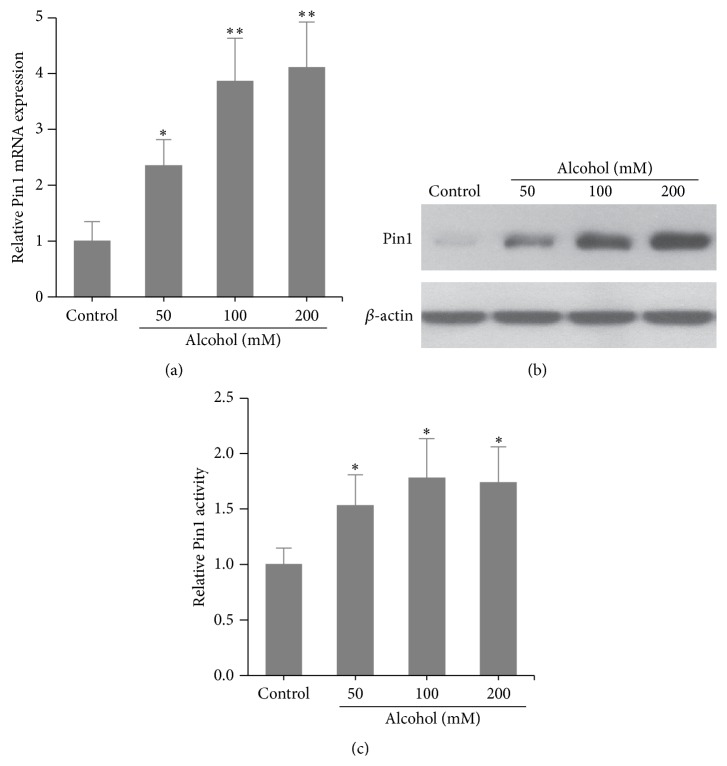
Pin1 expression and activity were upregulated by alcohol treatment of cardiomyocytes. (a) qRT-PCR of Pin1 mRNA expression in cardiomyocytes treated with alcohol (0, 50, 100, or 200 mM) for 24 h. (b) Western blot of Pin1 expression in cardiomyocytes treated with alcohol at indicated concentrations. (c) Pin1 activity assays in cardiomyocytes treated with alcohol at indicated concentrations. ^*∗*^ *p* < 0.05 and ^*∗∗*^ *p* < 0.01 compared to cells without alcohol treatment.

We had previously demonstrated that high doses of alcohol induce cardiomyocyte apoptosis [11]. To investigate whether Pin1 plays a role in alcohol-induced apoptosis in cardiomyocytes, we first analyzed Pin1 expression after exposing cells to different concentrations of alcohol (ethanol: 0, 50, 100, or 200 mM). As shown in Figures 1(a) and 1(b), alcohol caused dose-dependent upregulation of Pin1 expression at both the mRNA and protein levels. Moreover, alcohol increased Pin1 activity in a dose-dependent manner (Figure 1(c)).

### 3.2. Pin1 Regulated Alcohol-Induced Apoptosis in Cardiomyocytes

**Figure 2:**
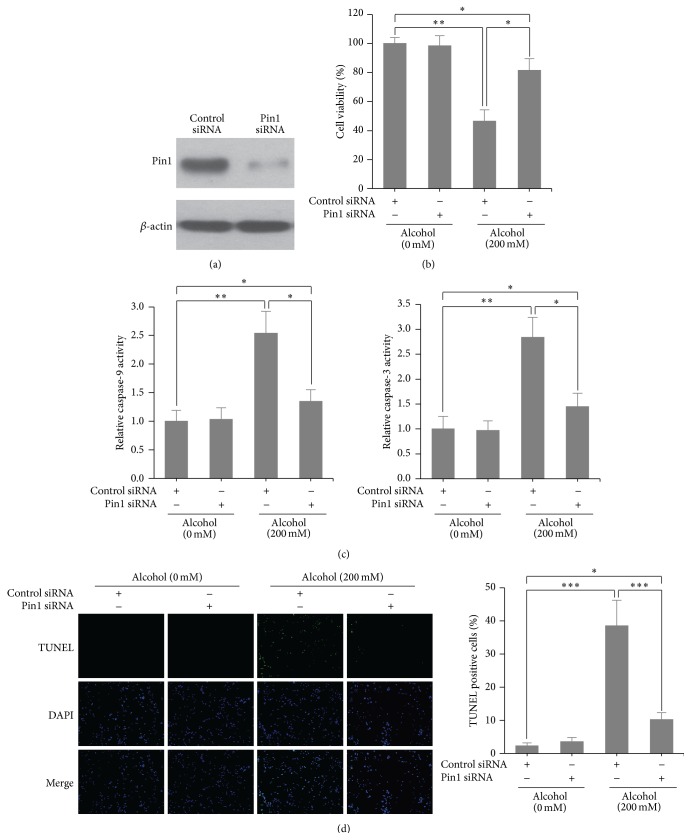
Knockdown of Pin1 inhibited alcohol-induced cardiomyocyte apoptosis. (a) Pin1 protein expression measured by western blotting after Pin1 siRNA transfection. ((b)–(d)) Cell viability assay (b), caspase-9 and caspase-3 activity assays (c), and TUNEL staining (d) in Pin1-knockdown cardiomyocytes treated or untreated with alcohol (200 mM) for 24 h. ^*∗*^ *p* < 0.05, ^*∗∗*^ *p* < 0.01, and ^*∗∗∗*^ *p* < 0.001.

**Figure 3:**
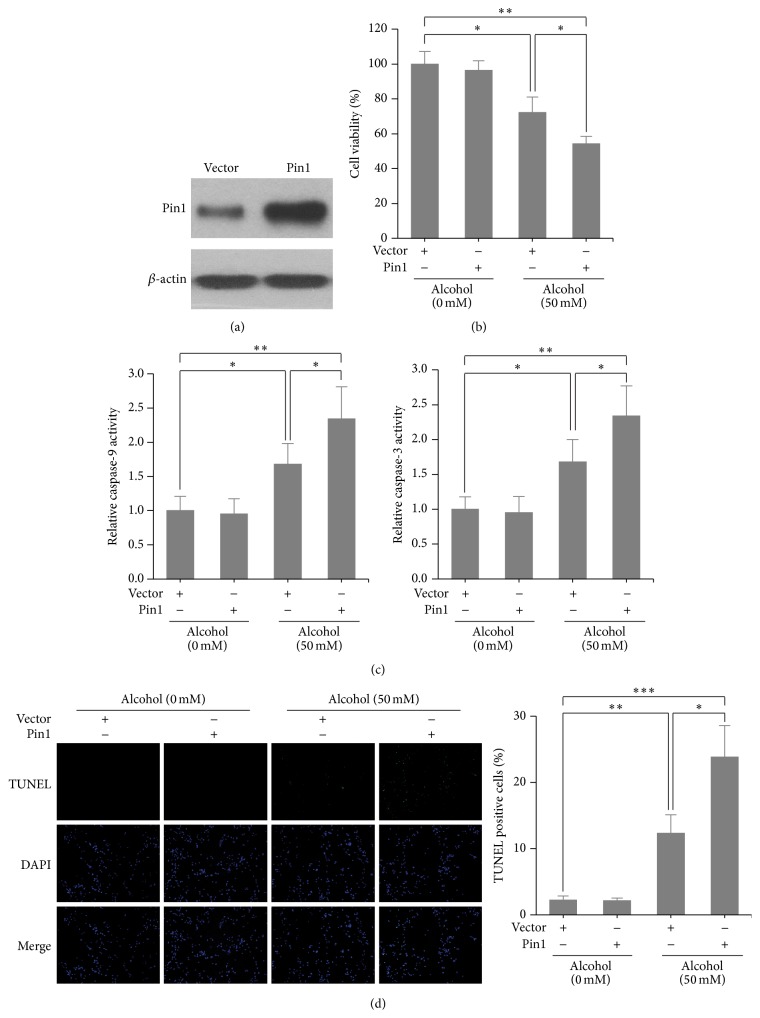
Overexpression of Pin1 enhanced alcohol-induced cardiomyocyte apoptosis. (a) Pin1 protein expression measured by western blotting after Pin1 plasmid transfection. Cell ability (b), caspase-9 and caspase-3 activity assay (c), and TUNEL staining (d) in Pin1-overexpressed cardiomyocytes treated or untreated with alcohol (50 mM) for 24 h. ^*∗*^ *p* < 0.05, ^*∗∗*^ *p* < 0.01, and ^*∗∗∗*^ *p* < 0.001.

We investigated whether Pin1 is involved in alcohol-induced cardiomyocyte apoptosis by both loss- and gain-of-function studies. We first transfected mouse cardiomyocytes with control or Pin1 siRNA for 24 h and exposed those cells to alcohol (200 mM) for another 24 h. Knockdown efficiency was confirmed by western blotting (Figure 2(a)). As expected, cell viability decreased by more than 50% in the alcohol-treated group as compared to cells in the control group. However, the viability of Pin1-knockdown cells only decreased by 20% with alcohol treatment (Figure 2(b)). Caspase-9 and caspase-3 activity assays and TUNEL staining consistently showed that alcohol-induced cell apoptosis was inhibited by depletion of Pin1 (Figures 2(c) and 2(d)). Interestingly, ectopic overexpression of Pin1 further enhanced alcohol-induced apoptosis and loss of cell viability (Figures 3(a)–3(d)). Together these results indicate that Pin1 plays an important role in promoting alcohol-induced apoptosis in cardiomyocytes.

### 3.3. Knockdown of Pin1 Reduced Alcohol-Mediated Mitochondria Oxidative Stress in Cardiomyocytes

**Figure 4:**
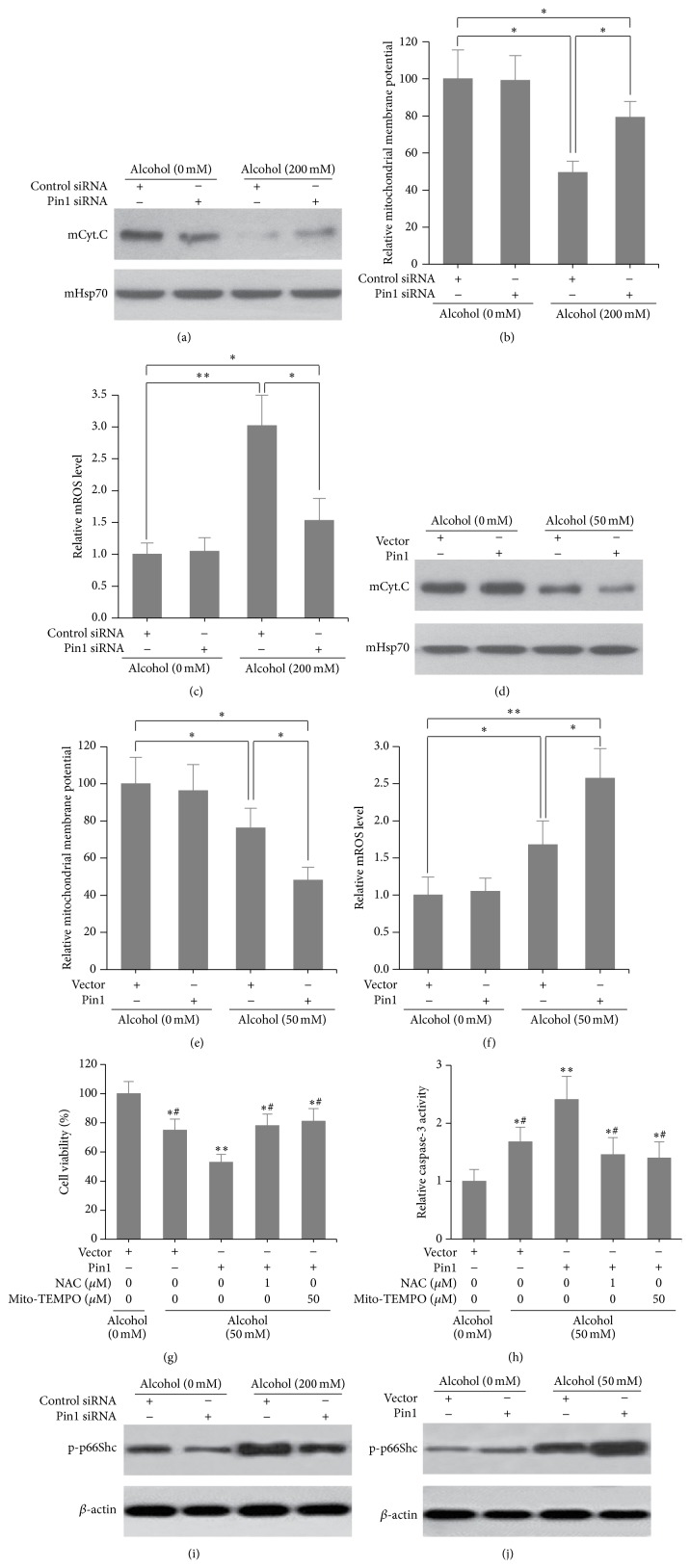
Pin1 enhanced alcohol-mediated mitochondria oxidative stress in cardiomyocytes. (a) Western blot of mCyt.C release in Pin1-knockdown cardiomyocytes treated or untreated with alcohol (200 mM) for 24 h. Relative mitochondrial membrane potential (b) and mROS levels (c) in cells are indicated. (d) mCyt.C levels in Pin1-overexpressed cardiomyocytes treated or untreated with alcohol (50 mM) for 24 h. Relative mitochondrial membrane potential (e) and mROS levels (f) in cells are indicated. ^*∗*^ *p* < 0.05 and ^*∗∗*^ *p* < 0.01. Cell viability (g) and caspase-3 activity (h) assays in Pin1-overexpressed cells treated with alcohol (50 mM) and NAC (1 *μ*M) or Mito-TEMPO (50 *μ*M). ^*∗*^ *p* < 0.05 and ^*∗∗*^ *p* < 0.01 compared with cells untreated with alcohol; ^#^ *p* < 0.05 compared with Pin1 overexpression cells treated with alcohol only. (i) Western blot of mitochondrial p-p66Shc levels in control or Pin1-knockdown cardiomyocytes treated or untreated with alcohol (200 mM) for 24 h. (j) Western blot of mitochondrial p-p66Shc levels in Pin1-overexpressed cardiomyocytes treated or untreated with alcohol (50 mM) for 24 h.

Mitochondrial oxidative signaling contributes to alcohol-induced apoptosis [11], and downregulation of Pin1 can prevent mitochondrial oxidative stress in patients with diabetes [23]. Thus, we investigated whether depletion of Pin1 can prevent alcohol-induced mitochondrial oxidative stress in cardiomyocytes. mCyt.C, mitochondrial membrane potential, and mitochondrial reactive oxygen species (mROS) were examined in control and Pin1-knockdown cardiomyocytes in the presence or absence of 200 mM alcohol. Downregulation of Pin1 significantly reversed mCyt.C release, reduced mitochondrial membrane potential, and stimulated mROS production induced by alcohol treatment (Figures 4(a)–4(c)). We next sought to recapitulate the result by using Pin1 inhibitor Juglone. Unlike Pin1 knockdown cells, cells treated with Pin1 inhibitor Juglone (2.5 *μ*M) demonstrated lower cell viability and higher apoptosis in both condition without or with alcohol treatment (Supplemental Figure 1; see Supplementary Materials available online at http://dx.doi.org/10.1155/2016/4528906). Since Pin1 has been reported to be a strong cytotoxic agent and induce apoptosis in many cell types [24, 25], the apoptosis-inducing activity of Juglone in cardiomyocytes might be through other signaling pathways than inhibiting Pin1. We next overexpressed Pin1 in cardiomyocytes. As expected, alcohol induced mCyt.C release, mitochondrial membrane potential reduction, and ROS production, which were further enhanced by Pin1 overexpression (Figures 4(d)–4(f)). In addition, we tested whether scavenging of ROC could reverse cell viability and apoptosis in alcohol-treated cardiomyocytes. As showed in Figures 4(g) and 4(h), two ROS scavengers (NAC and Mito-TEMPO) all partly rescued alcohol-induced cell death and apoptosis in Pin1-overexpressed cardiomyocytes. Our previous work has found that Pin1 interacts with p-p66Shc which translocates to mitochondria and functions as a redox enzyme to regulate cardiomyocyte apoptosis [11]. To further investigate the downstream signaling of Pin1 in alcohol-induced cardiomyocyte apoptosis, we analyzed the mitochondrial p-p66Shc levels in cardiomyocytes treated with or untreated with alcohol in Pin1 knockdown or overexpression cells. As we expected, knockdown of Pin1 reduced and overexpression of Pin increased mitochondrial p-p66Shc level (Figures 4(i) and 4(j)). Taken together, this demonstrates that Pin1 promotes alcohol-mediated mitochondria oxidative stress in cardiomyocytes.

### 3.4. Pin1 Regulated Alcohol-Mediated NO Production and eNOS Expression in Cardiomyocytes

**Figure 5:**
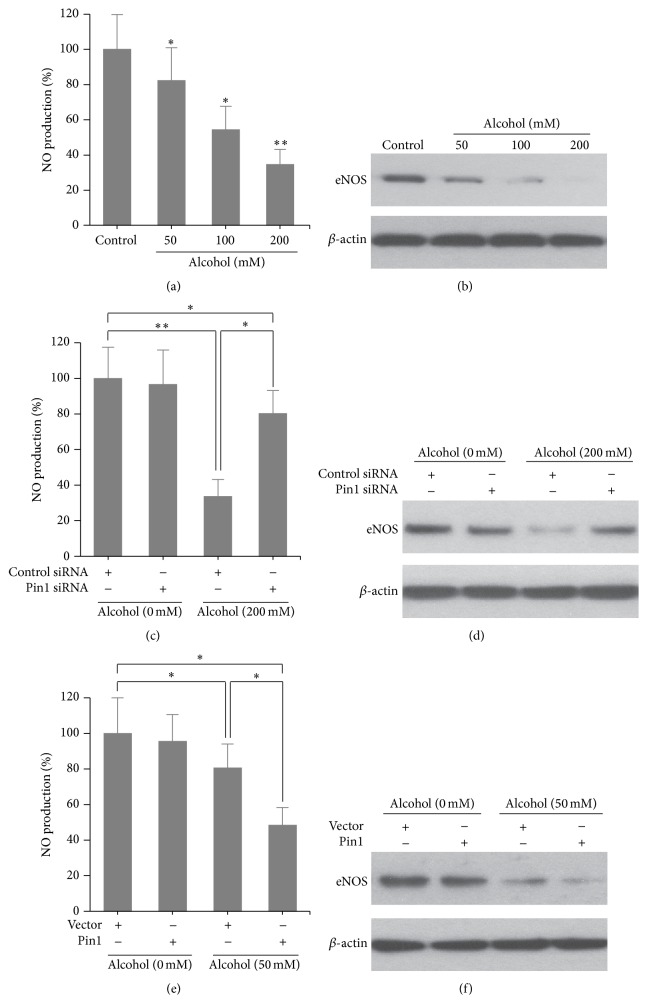
Pin1 reduced the NO production and eNOS expression that were inhibited by alcohol in cardiomyocytes. (a) NO production in cardiomyocytes treated with alcohol (0, 50, 100, or 200 mM) for 24 h. (b) eNOS expression analyzed by western blotting in cells treated with alcohol at indicated concentrations. NO production (c) and eNOS expression (d) in Pin1-knockdown cardiomyocytes treated or untreated with alcohol (200 mM) for 24 h. NO production (c) and eNOS expression (d) in Pin1-overexpressed cardiomyocytes treated or untreated with alcohol (50 mM) for 24 h. ^*∗*^ *p* < 0.05 and ^*∗∗*^ *p* < 0.01.

Since nitric oxide (NO) plays a role in apoptosis of cardiomyocytes [24], we investigated whether alcohol mediates endothelial eNOS expression. As shown in Figures 5(a)–5(c), alcohol inhibited NO production and eNOS levels in a dose-dependent manner. We also tested alcohol-induced NO production and eNOS expression in Pin1-knockdown cells. Compared to control cells, alcohol significantly decreased NO production, while knockdown of Pin1 partly reversed its decrease of production (Figure 5(c)). eNOS expression was consistently rescued by Pin1 depletion in the presence of alcohol (Figure 5(d)). To further confirm Pin1 function in alcohol*-*mediated NO production and eNOS expression, we overexpressed Pin1 in cardiomyocytes and assessed NO and eNOS levels after alcohol (50 mM) treatment. As shown in Figures 5(e) and 5(f), overexpression of Pin1 further inhibited NO production and eNOS expression, which demonstrates that Pin1 regulates alcohol-mediated NO production by affecting eNOS expression in cardiomyocytes.

## 4. Discussion

**Figure 6:**
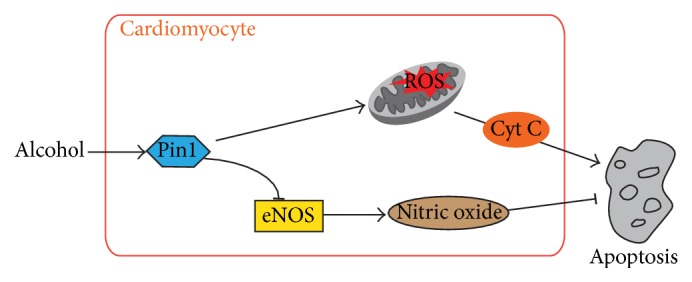
Schematic representation of Pin1 in the process of alcohol-induced cardiomyocyte apoptosis.

Apoptosis is a mechanism of programmed cell death implicated in the pathogenesis of alcohol-induced left ventricular dysfunction [1]. Several mechanisms have been proposed to explain the role of alcohol during development of ACM, such as upregulated ROS levels and decreased NO production [11, 25, 26]. In this study, we found that alcohol elevated Pin1 expression and activity in mouse primary cardiomyocytes. Pin1 in turn played a pivotal role in alcohol-induced cardiomyocyte apoptosis by promoting ROS accumulation and repressing eNOS expression (Figure 6).

ROS are free radicals containing oxygen molecules, mostly generated in mitochondria, which contain peroxyl, alkoxyl, superoxide anions, hydroxyl radicals, and oxygen derived nonradical species [27]. Depending on the concentration of ROS, it can be beneficial or harmful to cells and tissues [28]. Normal ROS metabolism plays an essential role in disease resistance and cell-mediated immunity. However, high levels of ROS may cause uncontrolled oxidation of lipids, proteins, and DNA, which finally leads to apoptosis [27]. Previous studies have suggested that high levels of alcohol can induce ROS-mediated apoptosis in cardiomyocytes [8, 11]. Since we found that alcohol treatment stimulated expression and activity of Pin1 in mouse primary cardiomyocytes, we then investigated the role of Pin1 in regulation of alcohol-induced ROS accumulation in mitochondria. Our results showed that depletion of Pin1 significantly reduced the mCyt.C release, loss of mitochondrial membrane potential, and mROS production stimulated by alcohol treatment of cardiomyocytes, which might be mediated by p66^shc^. Conversely, overexpression of Pin1 even enhanced cardiomyocyte apoptosis. Interestingly, the increase of Pin1 activity was not as high as the increase of Pin1 expression level when we treated cardiomyocytes with high-dose alcohol, which indicates that Pin1 might function by interacting with other cofactors.

We also found that Pin1 promoted alcohol-induced cardiomyocyte apoptosis by inhibiting NO production. NO is a free radical, the product of the reaction catalyzed by eNOS [29]. NO levels were originally found to be closely related to inflammatory status, and NO promotes tumor cell proliferation, mobility, and invasiveness [29–31]. With regard to apoptosis, Shen et al. reported that upregulation of eNOS protects cardiomyocytes from apoptosis [25]. By investigating regulation of NO production by Pin1, we demonstrated that knockdown of Pin1 rescued the decrease in eNOS expression and NO production, and ectopic overexpression of Pin1 resulted in less eNOS expression and NO production when the cells were treated with 50 mM of alcohol. However, without alcohol treatment, we did not observe significant changes in NO production by overexpression of Pin1. We suspect that the inhibitory effect of Pin1 on NO production is induced by alcohol. Therefore, the mechanisms by which alcohol regulates Pin1 expression and activity remain to be elucidated. Additionally, since NO and ROS scavenge each other, the effect of Pin1 on NO production may also be affected by alcohol-induced ROS accumulation [32, 33]. Pin1 has been reported to recognize Ser-116 eNOS inhibitory phosphorylation to impair NO release. Therefore Pin1 might regulate eNOS activity and NO production by directly interacting with eNOS [34].

In conclusion, our findings demonstrate that alcohol induces Pin1 expression and activation in a dose-dependent manner in mouse primary cardiomyocytes. We also found that depletion of Pin1 significantly inhibited cardiomyocyte apoptosis by regulating mROS accumulation and NO production. Thus, we found that Pin1 plays a pivotal role in alcohol-induced cardiomyocyte apoptosis, and Pin1 is a potential therapeutic target for left ventricular dysfunction caused by excessive alcohol consumption.

## Supplementary Material

Cardiomyocytes were treated with Pin1 inhibitor Juglone (2.5 μM). Cells treated with Juglone demonstrated lower cell viability and higher apoptosis in both condition without or with alcohol treatment. Since Pin1 has been reported to be a strong cytotoxic agent and induce apoptosis in many cell type, the apoptosis-inducing activity of Juglone in cardiomyocytes might be through other signaling pathways than inhibiting Pin1.
