# Supplementary figures and images for: Targeting Pin1 Protects Mouse Cardiomyocytes from High-Dose Alcohol-Induced Apoptosis
Source: Oxid Med Cell Longev. 2015 Dec 1;2016:4528906. doi: 10.1155/2016/4528906 (PMC4678095; doi:10.1155/2016/4528906)

(a)

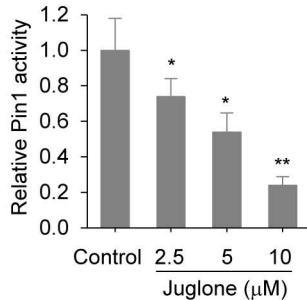

(b)

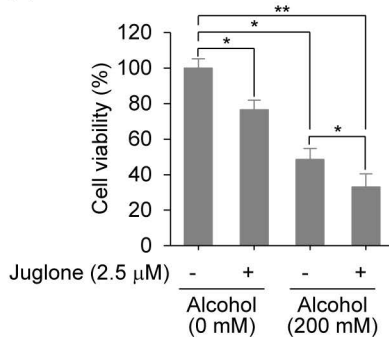

(c)

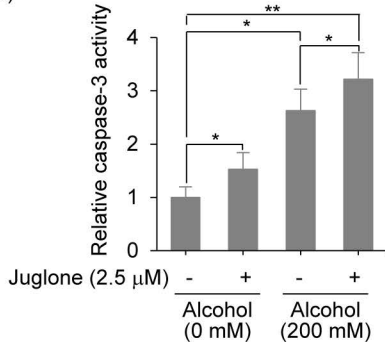

Supplement: Supplementary file 1 — Cardiomyocytes were treated with Pin1 inhibitor Juglone (2.5 μM). Cells treated with Juglone demonstrated lower cell viability and higher apoptosis in both condition without or with alcohol treatment. Since Pin1 has been reported to be a strong cytotoxic agent and induce apoptosis in many cell type, the apoptosis-inducing activity of Juglone in cardiomyocytes might be through other signaling pathways than inhibiting Pin1. [file 4528906.f1.pdf]
